# Incorporating Psychoeducational Care in the Autism Diagnosis Pathway: Experiences, Views, and Recommendations of UK Autistic Adults and Autism Professionals

**DOI:** 10.1089/aut.2023.0060

**Published:** 2025-02-05

**Authors:** Bryony Beresford, Suzanne Mukherjee

**Affiliations:** Social Policy Research Unit, School for Business and Society, University of York, York, United Kingdom.

**Keywords:** post-diagnostic care, psychoeducational care, autistic adults, psychoeducation programme

## Abstract

**Background:**

When someone receives a diagnosis they may need support with information and emotional needs. These are called psychoeducational needs. For adults diagnosed with autism, these can include needing to understand and make sense of the diagnosis and finding self-management strategies that work for them. When autistic adults do not receive the psychoeducational support they need their mental health and self-confidence in managing everyday life is affected. However, many diagnostic services do not provide psychoeducational care. In this study, we investigated autistic adults' and autism specialist staff's views on the psychoeducational care that diagnostic services should provide.

**Methods:**

We recruited 26 autistic adults and 30 staff working in 8 UK autism services commissioned to provide both diagnostic assessments and post-diagnostic care. The staff sample included five autistic adults employed as “experts by experience” to co-deliver psychoeducational support. We used group discussions (or, where required, 1:1 interviews) to explore their views and experiences.

**Results:**

Study participants believed psychoeducational needs arose during the assessment process (e.g., possible emotional reactions to diagnosis), and when the diagnosis is divulged (e.g., managing disclosure) as well as during the weeks and months following diagnosis. In this period, study participants agreed that the psychoeducational care offered by diagnostic services should include a debrief appointment, psychoeducation program, and the provision of “curated” information. That is, information resources carefully selected by staff and in multiple formats (e.g., text-based, videos). Study participants believed autism professionals and “experts by experience” had distinct contributions to make in meeting psychoeducational needs.

**Conclusion:**

Findings support the case for diagnostic services to have the resources to address psychoeducational needs across the diagnostic pathway, including the offer of a debrief appointment and group-delivered psychoeducational program (with the option for 1:1 delivery) post-diagnosis. “Experts by experience” should be integral to the development and delivery of psychoeducational care.

## Abstract (plain language summary)

Community brief

Why is this an important issue?

When someone receives a diagnosis, they may have needs for information and emotional support, called psychoeducational needs. In the United Kingdom (UK), most autism diagnosis services are not able to offer psychoeducational care following diagnosis. However, for some, being diagnosed as autistic can be emotional and unsettling. Some people may feel they know very little about autism or feel unsure about how to best manage everyday life as an autistic adult. It is important that we understand what autism diagnostic services should be doing to meet people's psychoeducational needs.

What was the purpose of this study?

Our aim was to develop recommendations for diagnostic services on how to meet the psychoeducational needs that can arise when someone is diagnosed as autistic.

What did the researchers do?

We spoke to 26 autistic adults living in the United Kingdom about their experiences of being diagnosed as adults and their views about how people should be supported in the days and months following diagnosis. We used online focus groups or, if requested, individual interviews to hear their views and experiences. We also used workshops to consult with 30 staff working in autism diagnosis services that also offered extended post-diagnostic support. The staff taking part included five autistic adults employed as “experts by experience” to codeliver psychoeducational support.

What were the results of the study?

Many autistic adults described difficult experiences around the time of their diagnosis, of feeling isolated and unsupported, or poorly supported. However, some had attended “post-diagnostic” groups, which they found very helpful for understanding and making sense of the diagnosis, learning self-management skills, and making connections with other autistic adults. Everyone agreed it was essential for diagnostic services to recognize and attend to the needs for information and emotional support which arise from going through an autism diagnosis. They argued that it should include the following: (1) guidance on disclosure at the assessment feedback appointment; (2) information resources in multiple formats (including autistic people's accounts of living with autism); (3) the offer of a debrief appointment; and (4) a post-diagnostic group (or 1:1 sessions). There was also strong agreement that professionals and autistic adults need to work together to design and deliver resources and group programs.

What do these findings add to what is already known?

Previous studies have highlighted the importance of post-diagnostic support. This is the first study that sought to specify how autism diagnostic services should meet the psychoeducational needs that can arise when someone is diagnosed as autistic.

What are the potential weaknesses in the study?

The majority of the autistic adults who took part in this study identified themselves as White British. The study was conducted in the United Kingdom and our findings and recommendations may not directly transfer to other countries.

How will these findings help autistic adults now or in the future?

Directly based on the study findings, we make recommendations that diagnostic services can use to develop or extend what they provide. Going forward, we need research that evaluates the impacts of providing high-quality psychoeducational care. This will help to make the case for diagnostic services to have the funds and resources to offer psychoeducational care following an autism diagnosis.

## Background

Timely access to diagnostic assessment is a priority for autistic people and professionals. Recent studies, however, highlight the importance of also paying attention to the information and emotional support needs *directly associated* with receiving an autism diagnosis. Wider evidence on the emotional and informational needs arising from any (significant) diagnosis supports this argument. Across diagnoses, these needs include: knowledge and understanding the condition (and, if relevant, its treatment); exploring emotional responses to the diagnosis; acquiring self-care and self-management strategies; managing sharing the diagnosis with others; and being informed about the formal and informal support available.^1–4^ Such needs are often collectively referred to as psychoeducational needs, a term that captures both the therapeutic and informational consequences of a diagnosis.^5,6^

Psychoeducational programs are interventions specifically designed to address psychoeducational needs. Such programs are condition-specific, manualized interventions, spanning a fixed number of sessions, which integrate educational elements and simple therapeutic work. They are delivered by a professional(s) with relevant qualifications and expertise who are based in (or work with) the service making the diagnosis. The objective of such programs is to educate, support acceptance, and empower newly diagnosed individuals and, in some cases, their families.^1,7,8^ Group, as opposed to 1:1 delivery, is the preferred mode of delivery as it brings the additional benefits of shared learning and connecting with others with the same experience.^9–11^ For some conditions (e.g. bipolar disorder, cancer, fibromyalgia), psychoeducational programs are often a routine part of the care pathway. For some diagnoses, psychoeducation programs may be codelivered by professionals and people with lived experience of the condition (referred to as experts by experience or peer mentors).

Although limited in its size and quality, current evidence across a number of different conditions indicates group-delivered psychoeducation programs are welcomed and meet the needs of those who attend them. There is also some evidence of their effectiveness in terms of knowledge and understanding of the condition, psychological and social outcomes, and adherence to treatments.^7,12–16^ Some studies have also examined their cost-effectiveness, with some concluding there is evidence of cost-effectiveness, although the evidence base on this is very limited.^17–19^

Alongside group programs, some self-guided, or self-directed, psychoeducation programs have also been developed for some diagnostic groups, although by their nature they cannot offer a substantive therapeutic element. The evidence on their effectiveness is more equivocal with evidence suggesting considerable individual differences in the extent to which people engage with them and assimilate and implement the knowledge acquired.^20^

Studies of the experiences and outcomes of an autism diagnosis paint a mixed picture. They suggest that for some receiving the diagnosis is, *in itself*, highly beneficial (e.g. bringing a sense of relief and validation, facilitating access to support), and supports wider positive outcomes being achieved.^21–24^ However, research also reports a range of difficult emotional reactions (e.g., confusion, anger, anxiety, grief, dismay, uncertainty) and unmet support needs around understanding and coming to terms with the diagnosis and managing everyday life, with studies calling for increased or improved support in the period following diagnosis.^21,23–31^ Such evidence suggests that failing to recognize and attend to such needs runs the risk of an autism diagnosis being an unhelpful, or even harmful, intervention that may threaten mental health and other outcomes.

Furthermore, evidence is beginning to emerge on the *benefits* of meeting psychoeducational needs arising from an autism diagnosis, including offering the opportunity to attend an autism psychoeducation program. Although, to date, no trials evaluating such programs have been conducted,^32^ a mixed-methods study has compared the outcomes and experiences of adults who did, or did not, attend a psychoeducation program.^21^ It found that accounts of the months following diagnosis differed between the two groups. Many of those who had not attended a psychoeducation program reported unresolved (and sometimes quite significant) difficulties around understanding and coming to terms with the diagnosis, with some attributing deteriorations in their mental health to this. This contrasted with the accounts of those who attended a program who typically believed it had wide-ranging and positive impacts, including greater self-acceptance and sense of connection with others, feeling equipped to manage everyday life and being able to see the strengths, or positives, of being autistic.

Alongside this qualitative evidence, a preliminary comparison of outcomes found differences in mental health, self-efficacy, and connections with autistic peers 6–9 months post-diagnosis favoring those who had attended a psychoeducation program.

Despite the growing evidence base on the psychoeducational implications of receiving an autism diagnosis, in the United Kingdom (UK) as well as other countries, most adults diagnosed with autism receive no or very limited psychoeducational care from diagnostic services. This is due, at least in part, by its omission from national clinical or commissioning guidance.^21,33^ If offered at all, psychoeducational care typically comes in the form of a single follow-up, or debrief, appointment. However, a small number of UK diagnostic services are commissioned to provide extended post-diagnosis support and, for almost all, this includes a group-delivered psychoeducation program (with most also offering the alternative option of 1:1 sessions). The absence of published programs has resulted in these services developing their own programs “in-house.”

Although there is some common content, these programs also differ in a number of ways; for example, duration, group make-up and size, teaching and therapeutic approaches used, the professionals involved, and the involvement of experts by experience.^21^

This article reports a study that sought to specify the psychoeducational care that autism diagnostic services should provide through consulting with and drawing on the perspectives and experiences of autistic adults, experts by experience and professionals working in specialist autism services. (We note that this research was concerned with the psychoeducational care *autism diagnostic services* should provide. Other types of support that people may draw on following an autism diagnosis, such as support groups led by autistic adults, can also play a role in meeting information and support needs post-diagnosis.^34,35^)

## Methods

### Study design

As is often the case in applied health research, we used a generic qualitative approach.^36,37^ That is, our study design was not situated within a particular methodological perspective (e.g., grounded theory, narrative approach). Rather, we used qualitative methods to explore and elicit study participants' views and perspectives.

The target sample comprised: people diagnosed as autistic in adulthood; professionals based in specialist autism services providing diagnostic assessment and post-diagnostic support, including psychoeducation programs; and individuals employed by these services to act as experts by experience (or peer mentors) for the psychoeducational program.

A sequential design was used in which data collection with autistic adults preceded data collection with diagnostic assessment service staff. This allowed selected findings from the research with the autistic adults to be used to stimulate reflection and discussion in the focus groups held with staff.

Focus groups were used to explore views and experiences and elicit recommendations regarding psychoeducational care. Autistic adults who did not feel able to participate in a focus group because of the stress/anxiety this would cause were offered an individual interview instead. Data collection took place between April and June 2021. Study participants were invited to review and feedback on a draft report of the findings and recommendations.

The operational team that commissioned and oversaw this research included representatives from a national autism charity. It also had a permanent advisory group of experts by experience, including autistic adults. At key stages in the project, the researchers consulted with this team via online meetings and email discussions regarding the scope and design of the work, their views and reflections on emerging findings, and the service delivery and practice implications being drawn. The study was approved by a University of York Departmental Ethics Committee (Ref.: SPSW/S/21/1). The researchers (B.B., S.M.) are neurotypical, applied health and care service researchers.

### Sampling and recruitment

#### Autistic adults

The aim was to recruit individuals diagnosed by a number of different autism diagnostic services in the UK, and for the sample to include those who had attended a psychoeducation program and those who had not. No upper age limit was imposed, nor time since diagnosis. A pragmatic approach to recruitment was used, with study invitations distributed by two diagnostic services (both of which offered a psychoeducation program), a national autism advocacy organization, and via the researchers' existing public involvement networks.

#### Professionals and experts by experience

Nine autism specialist services commissioned to provide diagnostic assessment and post-diagnostic support were invited to take part. All had been identified by a previous study.^21^ Eight agreed and the service/senior lead identified team members with direct experience of diagnostic assessments or delivering the psychoeducation program, and shared recruitment packs with them. In services where the psychoeducation program was co-delivered by one or more experts by experience, the service/senior lead also shared recruitment packs with these individuals.

All study participants completed an online consent form before taking part.

### Study sample

Twenty-six autistic adults, 5 experts by experience and 25 professionals were recruited. The autistic adults (aged 21–64 years [median 39 years]) included 13 self-identifying as male and 9 as female. The remainder reported another gender identity (small numbers preclude detailed reporting). The majority (*n* = 24) described themselves as White British. Time since diagnosis ranged from 1 to 26 years (median 6 years) and multiple autism diagnostic services were represented in the sample. Ten had attended an autism psychoeducation program, with five different diagnostic services represented in this subgroup.

Experts by experience were recruited from two of these services and had been working in this role between <12 months and 9 years. The professions represented in the autism professional sample included: clinical psychology; mental health nursing; occupational therapy; speech and language therapy; and social work. An overview of the psychoeducational programs delivered by participating services is provided in Supplementary Material S1.

### Data collection

Data collection (focus groups, interviews) was conducted remotely by video-conferencing software. The researchers (B.B., S.M.) ran the focus groups together. One researcher (S.M.) carried out the interviews. All were audio-recorded. Focus group/interview topic guides are presented in Supplementary Material S2.

#### Autistic adults

Most autistic adults took part in a focus group. Two chose an individual interview. Each focus group was only attended by others recruited via the same recruitment pathway. They lasted 90 minutes, with a 10-minute break halfway through. Focus groups and interviews explored views and experiences regarding: the diagnostic assessment process; information and support needs directly arising from the diagnosis; information and help-seeking in the post-diagnosis period; types of psychoeducational care that diagnostic services should provide, and how it should be provided; and group-delivered interventions, including strategies to support participation.

#### Experts by experience and autism service professionals

Experts by experience and professionals attended a half-day workshop-style event that incorporated two focus group discussion sessions (35 and 50 minutes, respectively). Both were preceded by a brief presentation of findings from the research with autistic adults. The first discussion explored views about psychoeducational needs during the assessment process and feedback appointment, including strategies and practices used to address these, and views how these could be better met. The second discussion focused on psychoeducation programs and explored: advantages and disadvantages of using groups for psychoeducation interventions; personalizing group-delivered psychoeducational programs; the role(s) played by experts by experience, and their training and support needs; and supporting engagement with/attendance of group delivered programs. Owing to the numbers attending, workshop participants were split into two groups for the discussions. The workshop was run twice. On each occasion, four different specialist autism services were represented.

### Data analysis

Data analysis was conducted in two stages. First, data on autistic adults' experiences of being diagnosed was analysed so that we could share these findings at the workshops with staff. (An account of the findings of this analysis is presented elsewhere.^38^) The second, and main, round of analysis took place once data collection was complete. Here the objective of data analysis was to synthesize the experiences, views, and recommendations of study participants in order to identify and specify the components of psychoeducational care that study participants believed should be core features of autism diagnosis pathways.

For both rounds of analysis, we used a thematic approach.^39^ The thematic frameworks developed including *a priori* (e.g., management of the feedback appointment; views on psychoeducation programs and their content) and emergent (e.g. experiences of unsupported information searching and disclosing diagnosis to others, supervision of experts by experience) topics or issues.

To start, structured, extended summaries (including verbatim quotes) of each focus group (and the two interviews) were created by one of the researchers (S.M.) through repeated, close listening to the audio-recordings. These replicated the structure of the focus group/interview topic guides. Drafts of these summaries were scrutinized by the second researcher (B.B.) before being finalized.

Next, the researchers (S.M., B.B.) reviewed the summaries separately and then together to identify data relevant to .understanding: i) the times or moments when psychoeducational needs may arise and should be attended to across the diagnostic pathway (e.g., at the start of the assessment, the feedback appointment, the sharing of the diagnostic assessment), ii) views on how diagnostic services should preempt or respond to these needs, both in terms of the types of intervention offered (e.g., debrief appointments) and iii) aspects of practice (e.g., presenting autism as a difference vs. a difficulty) and service delivery (e.g., delivering psychoeducation programs online vs. in-person). Written commentaries (including verbatim quotes) were then created on each of these topics/issues, collating all relevant data from the extended summaries. Within these commentaries, we took care to note differences in view between (and within) the three subsamples (i.e., autistic adults, experts by experience, professionals) and evidence of consensus within group discussions. Both authors contributed to developing, reviewing, and refining these commentaries.

A draft version of the findings and recommendations were circulated as an email attachment to study participants inviting feedback and comments. Six participants responded (three autistic adults, three professionals) with revisions made in light of feedback received (e.g. further details on the pros and cons and group delivery).

In reporting the findings, we used verbatim quotes to illustrate the points being made in the commentary. Quotes were attributed to research participants according to their unique study identification (ID) code with the following used to denote the three study participant groups: AA = autistic adult, EbE = expert by experience, PR = autism professionals.

## Findings

**FIG. 1.**
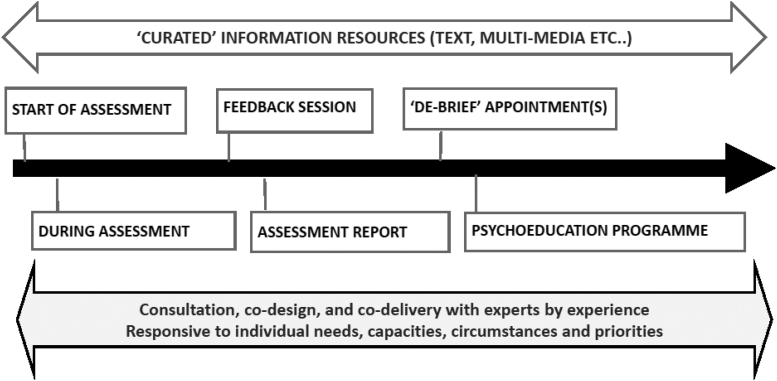
Psychoeducational care across the autism diagnosis pathway.

Participants believed psychoeducational needs can arise during the diagnostic process as well as in the post-diagnosis period. In this section we present their views about the nature of, and the rationale for, psychoeducational care across the diagnostic pathway (Fig. 1).

We start with reporting findings regarding the information resources that study participants believed should be offered (Fig. 1) alongside and in addition to psychoeducational care delivered through direct contact between autistic adults and staff.

### Provision of curated information

Autistic adults highlighted the risks associated with individuals independently seeking out information, particularly in the time immediately following diagnosis. This was because of the preponderance of false or out-of-date information, and the risk of being exposed to unhelpful perspectives.

[Services] need to be clued up enough to point people in the right direction before they go off and do their own thing and bump into these obscure websites and forums and people spreading misinformation. (AA11)

Some of the books I found were terrible, so I had to work out myself what was good (AA41)

In response to these experiences, they stressed the importance of diagnostic services curating^40^ the information resources they provide or recommend. In other words, the collection of information resources should be: (1) purposively selected to ensure that, together, they cover all required topics, and (2) carefully scrutinized and reviewed in terms of their quality and suitability.

The information study participants believed additional information resources needed to cover included: factual information about autism, formal and informal sources of support (local and national), and living with autism. “Living with autism” information resources regarded as most helpful were “life story/lived experience materials” materials and hints and tips on living with autism authored by autistic adults. Autistic adults were clear that information resources should be offered in a range of media—online and print, audio and video, and text and pictorial formats (e.g., comic book style).

The most useful thing was watching TED talks that dealt with how people deal with it on a daily basis. (AA31)

It's the “how to manage situations” that I didn't know and that's harder to find. You can only get this from other people on the spectrum. (AA32)

As with other components of psychoeducational care, autistic adults stressed that autistic adults (e.g., experts by experience working in the service) should be closely involved in selecting materials. Furthermore, recommended information resources should be regularly reviewed and updated where necessary.

### Psychoeducational needs and care: from assessment to feedback appointment

#### Assessment sessions

There was widespread agreement that the diagnostic assessment process itself generates psychoeducational needs. Autistic adults reported having been confused or puzzled by aspects of the assessment. Importantly, there were instances where the lack of explanation had affected acceptance of the diagnosis, or had made the process of making sense of it more difficult. The use of deficit, as opposed to difference, language during the assessment process was also regarded as influencing responses to the diagnosis.

You have to go through this process where they are telling you your deficits, it's essentially a very negative process, so they're telling you why you're failing at being neurotypical and then they say “Ok, that's why” and then that's it. It's upsetting. (AA42)

Finally, professionals believed explaining potential emotional and psychological reactions to receiving an autism diagnosis early in the assessment process helped individuals prepare for receiving a diagnosis.

#### The feedback session

All study participants believed the outcome of the diagnostic assessment should be shared in an appointment and, ideally, in-person. It was regarded a key point at which psychoeducational needs could be expressed, identified, and responded to.

They just emailed it to me and that was it. (AA32)

[It is important that we] can bear witness to the reaction and validate experiences. (PR20-Service D)

Furthermore, there was agreement that how the feedback session is managed can strongly influence how an individual reacts and responds to the news of the diagnosis. Three issues were highlighted. First, the explanation of why a diagnosis of autism had been reached should explicitly refer to findings from the different elements of the assessment process.

[The feedback appointment] didn't go through “this is what we found and this is why have decided to diagnose you as autistic.” And so I didn't help me to process it. (AA15)

Second, autistic adults strongly believed that the feedback appointment must include advice around disclosure. A number recounted very difficult experiences following disclosure and expressed regrets about the way, or with whom, they had shared news of the diagnosis. We note that, advice on disclosure was something that professionals had not typically previously considered, or routinely integrated into feedback appointments, apart from advice on notifying employers.

… there was an issue with safety for me after diagnosis as I told everyone, including people that didn't need to know. I could have done with some support straight after with knowing how to step out into the world. … how to step out of this room. I learnt a bit too late that you don't need to tell anyone, or maybe [just] to a few people. (AA26)

Finally, there was agreement among study participants that it was very important to pay attention to the language and terms used by staff, with terms presenting autism as a difference as opposed to a deficit preferred. However, autistic adults differed on whether the diagnosis should be presented as something positive, and whether struggles and difficulties should be acknowledged. Autism professionals and experts by experience believed a neutral position needed to be taken given the range of reactions that may be experienced.

### Psychoeducational needs and care: the post-diagnosis period

Study participants believed that all autism diagnostic services should offer a debrief appointment(s) and psychoeducational program. They also identified the assessment report as playing a role in meeting psychoeducational needs in the post-diagnosis period. Finally, they stressed the importance of providing or supporting access to additional information resources during this time (see Provision of curated information).

#### The assessment report

All the services represented in this study provided individuals with a detailed report of the diagnostic assessment. From the professionals' perspective, its main purpose was to support requests for adjustments or accommodations. For this reason, reports took a particular tone and emphasis, detailing difficulties and challenges. In contrast, the autistic adults believed *they* were the primary audience. They described finding the negative wording dispiriting and discouraging and undermined any positive rhetoric used during assessment and feedback sessions. The physical nature of the report increased the likelihood of individuals repeatedly returning to and dwelling on its contents.

When I got my letter it highlighted all the negative and none of the positives and it felt difficult to be left with that … (AA25)

Once professionals were made aware of this discrepancy in understanding, there was agreement for the need for clarity about the purpose of the report. The suggestion that assessment reports should incorporate as section on strengths was also widely supported.

#### Debrief appointment(s)

All participants believed that those receiving an autism diagnosis as adults should be offered at least one debrief appointment following diagnosis. This was because the feedback appointment was often an overwhelming experience that limited the extent to which questions and feelings could be explored. A debrief appointment (with, ideally, the option to offer more if required) was identified as providing an opportunity to explore why the diagnosis had been reached, initial emotional reactions, and sense-making. There was agreement that debrief appointments should be with the/a clinician involved in the diagnostic assessment.

It's really important that … there's a chance to sit down with somebody to discuss it, how you are feeling about it, and how you can move on from that point. (AA11)

Professionals working in services offering debrief appointments and psychoeducation programs believed the former addressed needs that group-delivered psychoeducation programs might not necessarily meet effectively. Furthermore, they believed debrief appointments supported take-up and readiness for the psychoeducation program, and ensured that maximum benefit was gained from attending it.

So, for me, I think going straight into a group isn't a good idea. (PR12-Service C)

There was agreement that time was needed between feedback and debrief appointments to allow for processing, and that individuals are likely to vary in terms of “ideal” timing. Offering a debrief appointment within a month of the feedback appointment, but with the option to delay, was generally regarded as a sensible approach.

#### Psychoeducation programs

A key study objective was to explore views about offering a group-delivered psychoeducation program in the post-diagnostic period. Overall, study participants agreed that all adults diagnosed as autistic should be offered this but services should have the option, where required, to deliver it via 1:1 sessions. In this section we report their views and recommendations on the design and delivery of psychoeducation programs for autistic adults.

##### Program content

Psychoeducation programs are defined as attending both to educational and therapeutic needs and, broadly speaking, program content is the same across diagnoses. Discussions with study participants about what should constitute the core curriculum of autism psychoeducation program aligned with these general principles and were as follows:

- Factual information about autism (e.g., diagnostic criteria; neurodiversity)
- Understanding and identifying how autism impacts me as an individual, and what it means for my life
- Sharing and exploring emotional responses to the diagnosis
- Learning self-management skills, particularly managing anxiety, disclosure, self-care, self-advocacy, and sourcing trustworthy information
- Information and advice on rights and entitlements
- Supporting connections with other autistic people.

Hard copies of teaching materials (e.g., Powerpoint slides) were strongly supported. Staff in services using “workbooks” (containing teaching materials as well as space for notes and recording individual responses to reflective activities etc.) reported they were well-received.

##### Group delivery

Group delivery is the recommended mode of delivering psychoeducation programs. Given groups can be particularly challenging for some autistic people, the issue of group delivery was carefully explored.

Among those without experience of group-delivered psychoeducation, some were very supportive of the idea because it afforded the opportunity to meet with other autistic people.

That would have been really helpful, to have prevented future stress. I would have found it really, really helpful to have met people in the same boat. (AA14)

Others, however, were ambivalent about, or opposed to, group-delivered support. Some questioned whether group-delivered interventions could ever offer therapeutic benefit for autistic people. There was also mistrust or skepticism that groups offered greater benefit than individual sessions, including concerns that individual needs would remain unaddressed. Some regarded group interventions as “cost-cutting” measures and were therefore devaluing.

I would feel fobbed off … and it feels like it's just a cheap way to offer it. … it's not tailored to me, it's quite general. It would work for some people, it would piss me off. (AA15)

In contrast, all the autistic adults who had attended a group-delivered psychoeducation program highlighted the importance and value of group delivery, a view shared by professionals and experts by experience. A range of particular benefits of group delivery were identified including: the opportunity to learn and share strategies from others living with autism; feeling understood and a sense of belonging; being in a social situation without feeling the need to mask; and validation from the group supporting self-acceptance.

It helped me to accept that being myself is OK. (AA21)

… there's been a massive positive shift in my mental health. … it's the bouncing ideas and thoughts off people, getting a different perspective, validation from group members. (AA51)

##### Co-development and co-delivery by professionals and experts by experience

All professionals and experts by experience believed psychoeducation programs should be codeveloped and codelivered. Some autistic adults (without direct experience of a psychoeducation program) questioned whether professionals needed to be involved. However, other autistic adults, as well as experts by experience and professionals, identified the unique knowledge, skills, and experience brought by professionals. These included: authoritative scientific knowledge about autism, extensive experience of the different ways autism can be manifested, and specific, specialist professional expertise in supporting autistic adults (e.g., clinical psychology, social work, occupational and speech and language therapies). In addition, they were equipped and qualified to identify (or manage disclosure of) risk, or the need for additional support. Their professional training also meant they were highly skilled in facilitating groups and using appropriate therapeutic techniques.

… while we get positive feedback for experts by experience, some people also want the authoritative knowledge of experts in the field, clinical psychologists. (EbE1)

… people can get upset and walk out or have disagreements, so you need to be cautious about what you are asking [experts by experience] people to take on. (PR-Service A)

We've had instances where people disclose risk information to the peer mentor and they [peer mentor] needs help in sorting this out. (PR-Service B)

Experts by experience were consistently identified as making two key and unique contributions as co-facilitators: authenticity and connection.

It's not necessarily in the content of what is being delivered but in the authenticity of the knowledge … and knowing that somebody actually identifies with the issue, and a feeling of trust that you are not being perceived as other. (AA25)

Among the services involved in this project, the terms under which experts by experience were contracted, or employed, to work with services was variable. Professionals noted that commissioners should be prepared to pay experts by experience at appropriate rates. Experts by experience and professionals both highlighted the importance of adequate training and support (e.g., debriefs and supervision).

I've spent a lot of time after debriefing because it can be very difficult for an individual. The group can bring up things for them as well, so it's about making sure they feel supported throughout. (PR28-Service B)

##### Program structure

There was strong consensus that didactic teaching should be minimal. The use of lived experience materials (e.g. short films) alongside factual information was strongly endorsed by autistic adults. Some services represented had revised their programs to decrease the taught content, and increase time allocated to interactive learning and the therapeutic elements of the program. Professionals from these services believed this had allowed greater personalization of the program content to reflect and respond to the characteristics, needs and concerns of group members. This was something identified as important by the autistic adults who took part in the study.

##### The presentation of autism

There was a range of views on how psychoeducation programs should present an autism diagnosis. Some autistic adults believed the diagnosis was something to be celebrated, others did not. Professionals and experts by experience observed that individuals can have very different feelings about their diagnosis, or be at different points in the process of processing or accepting it. They were unanimous that a neutral stance was required to hold the group together and ensure no one felt disenfranchised.

… this can be one of the most contentious things we deal with … we've had people storm out of sessions, people in tears, because it is one of the most emotionally charged things. People don't like being led in a particular way so it's just about validating people's personal feelings, it's just about emphasising it's okay to not be okay and it's okay to be okay. (EbE2)

##### Timing

Study participants agreed that the ideal time to attend a psychoeducation program varied between individuals. However, there was agreement that some time needs to elapse to allow for processing of the news of the diagnosis and its implications.

… it can take you to thinking about things you have never thought about before. … I think offering [psychoeducation programme] straight away might not be the best way forward, it needs to be flexible for the individual. (AA27)

I think it can tarnish the whole diagnosis experience if it's got wrong. … piling information too soon can be damaging because it can be overwhelming, they can't take it in, they just need a bit of breathing space to decompress and then they can take on a little bit more learning. Getting it wrong can put people off the service altogether, because they think that bit wasn't right for them so none of the service is going to be right for them. (EbE2)

For services represented in the study, the number of people being diagnosed typically determined the frequency at which programs are offered. Autistic adults noted the importance of informing people about likely wait times. Some who had experienced waiting described worrying that they had been forgotten. To avoid this, low-cost keeping in touch contacts (e.g., occasional emails) from services were suggested. Likewise, where an individual did not feel ready to attend a program, there was widespread support for services being able to re-offer the program on at least one further occasion.

##### Group size and make-up

Services represented in the study adopted different approaches to maximum group size, with this ranging from 8 to 12. Autistic adults consistently favored smaller sized groups.

There was 15 in my group and it was too much. Negotiating turn taking was difficult, stressful. (AA33)

Those running psychoeducation programs and autistic adults believed the value and benefit gained from group delivery could be compromised if an individual's age, gender, or sexuality were different from the majority in the group. Professionals noted the tension between offering the psychoeducation program in a timely way versus waiting until there were sufficient number to, for example, offer the option of attending a women-only group, or groups according to people's age.

From my perspective [woman 50+ years] group work would be better with not so much of a mix of ages. For example, one session was on relationships and the lads were laughing about it, giggling and one was talking about Tinder and they thought it was great fun, and I just thought I'm wasting my time here. (AA32)

##### Venues and time of day

Autistic adults stressed the importance of offering daytime and evening groups. They also urged services to use venues easily accessible by public transport and which could accommodate potential sensory needs. They noted that services did not need to use the service's main premises. Some had attended programs delivered in familiar, community venues and spoke very positively about these settings.

##### In-person versus online groups

The COVID pandemic meant services represented in this study had moved to online delivery of their psychoeducation programs. Study participants believed online delivery minimized the strain and emotional demands on individuals.

I'm probably more comfortable online, my anxiety is less sitting at home with a weighted blanket in my environment, so I'd rather get to know somebody that way when I'm having to suddenly meet people I don't know. (AA42)

[We] ran our first online group earlier this year and it was really successful in that people who wouldn't normally contribute to the group because they were a bit quiet were actually feeding in a lot more (EbE3)

However, among professionals and experts by experience there was caution and concern about online delivery. This was located primarily around experiences of lower, or skewed, take-up, and a reduced likelihood that on-going connections between group members will be formed. Professionals also noted that the one-to-one informal conversations that naturally happen during breaks or after in-person sessions did not happen in the online format. They believed this increased the risk for unmet needs remaining unidentified.

## Discussion

This article reports findings from research with autistic adults, and professionals and experts by experiences in autism diagnosis services, about psychoeducational needs and the psychoeducational care that autism diagnostic services should provide. It was carried out in response to growing evidence on the information and support needs associated with receiving an autism diagnosis, and the potential impacts if these needs are not met.^21,23–31^ This issue is not unique to autism. However, in other conditions greater attention has been paid to this aspect of post-diagnostic care. Indeed, psychoeducational interventions are regarded as a core element of post-diagnosis support for *parents* of children newly diagnosed as autistic.^41^ In contrast, current UK clinical guidance for autistic adults does not explicitly refer to psychoeducational care^42^: something that appears to be the case in other countries too.^33^ The objective of this study was to develop recommendations on the psychoeducational care of adults who are diagnosed as autistic.

Study participants were unanimous that multi-component, psychoeducational care should be provided by adult autism diagnosis services including the offer to debrief appointment(s) and psychoeducation program, alongside a well-constructed assessment report and access to curated sources of information. Furthermore, as well as offering psychoeducational care following diagnosis, attention should be paid to psychoeducational needs arising during the assessment period and at the feedback appointment. This in itself may also reduce or prevent the intensity of psychoeducational needs later on. Drawing the findings together, we offer four “high-level” recommendations:

- Diagnostic services should have the resource, skills, and capacity to address psychoeducational needs across the diagnostic pathway
- Psychoeducational care should include the offer to attend a psychoeducational program, with this typically delivered via groups, but with the option for 1:1 delivery. The psychoeducational program should be codesigned and codelivered with experts by experience.
- Curated information resources should support direct psychoeducational care. Experts by experience should be closely involved in the selection of material.
- Experts by experience involved in psychoeducational care should be carefully selected, trained, and supported. Pay levels/reimbursement of experts by experience should reflect their role and contribution.

We have also developed a detailed checklist of recommendations for autism services wishing to develop or extend their psychoeducational care, or review existing practice (see Supplementary Material S3). As noted earlier, to date there have been no independent, rigorous evaluations of existing psychoeducation programs.^32^ However, in the UK at least, there are a handful of diagnostic services that have developed, refined and delivered psychoeducation programs over a number of years.^21,38^ Such programmes may be a useful starting point for services wanting to offer a psychoeducational program as part of post-diagnosis care.

In recent years the value and importance of involving experts by experience in psychoeducation interventions has gained traction.^43–45^ Thus the intervention model can be regarded as shifting from interventions delivered by professionals to interventions co-developed and co-delivered by professionals and experts by experience. In this study, the distinct and different contributions of professionals and experts by experience were clearly articulated. However, as the professionals who took part in this study stressed, it is essential that experts by experience are trained and supported in their role, recruitment, and selection is deliberative and carefully undertaken, and levels of pay commensurate with the role.

### Study limitations

Overall, the sample successfully fulfilled the sampling frame. Among the autistic adults, there was a good mix of individuals with and without previous experience of participating in autism research and consultation work, different experiences of post-diagnostic support, and age. However, they were predominantly male or female and White British. This may limit the extent to which findings can be regarded as relevant to all those who receive a diagnosis of autism as adults.

### Directions for future research

Robust evaluations of psychoeducation programs are urgently required, including cost-effectiveness evaluations. Programs evaluated should be those developed (or updated) in consultation with autistic adults and codelivered by professionals and experts by experience. Research comparing mode of program delivery (in-person vs. online vs. blended) is also needed. A further priority is research that supports the development of good practice guidance on involving and working with experts by experience.
