# Supplementary material for: Incorporating Psychoeducational Care in the Autism Diagnosis Pathway: Experiences, Views, and Recommendations of UK Autistic Adults and Autism Professionals
Source: Autism Adulthood. 2025 Feb 5;7(1):13–24. doi: 10.1089/aut.2023.0060 (PMC11937777; doi:10.1089/aut.2023.0060)
Supplement: Supplementary Material S1 [file aut.2023.0060_suppl_materials1.docx]

**Supplementary Material #1:**

**Overview of key features^1^ of psychoeducation programmes delivered by services represented in the study**

| **ID** | **No.**  **sessions** | **Session duration** | **Group size** | **Facilitators** | **1:1 also available?** |
| --- | --- | --- | --- | --- | --- |
| A | 8 | 2 hrs | 8-12 | Clinical psychologists/ trainees  Occupational therapists (specific session(s)) | Yes |
| B | 11 | 2 hrs | 5-8 | Clinical psychologists/ trainees  Experts by experience | Yes |
| C | 13 | 2 hrs | Not available | Clinical psychologists/ trainees  Experts by experience | No |
| D & E | 5 | 1.5 hrs | 10 | Clinical psychologists/ trainees  Occupational therapists (specific session(s)) | No |
| F | 5 | 1.5 hrs | Not available | Clinical psychologists/ trainees  Occupational therapists (specific session(s)) | Yes |
| G | 6 | 2 hrs | 6-8 | Occupational therapists  Social workers  Experts by experience | Yes |

1. Programmes all comprised similar core content with that including: ‘feeling about diagnosis, what is autism, anxiety and its management, supporting work/study, sensory processing/sensory sensitivities, communication/social skills and relationships, and informal and formal support available. Some programmes also included substantive work on other mental health difficulties (e.g. depression), and programmes also varied in terms of the degree to which they were located in a wider neurodiversity perspective. Programmes varied in the balance between didactic teaching vs group work vs self-reflection. Overall, there had been reduction in the time allocated to didactic teaching.
