# Supplementary material for: Incorporating Psychoeducational Care in the Autism Diagnosis Pathway: Experiences, Views, and Recommendations of UK Autistic Adults and Autism Professionals
Source: Autism Adulthood. 2025 Feb 5;7(1):13–24. doi: 10.1089/aut.2023.0060 (PMC11937777; doi:10.1089/aut.2023.0060)
Supplement: Supplementary Material S2 [file aut.2023.0060_suppl_materials2.docx]

**Supplementary Material #2:**

**HEADLINE TOPIC GUIDES^[[1]](#footnote-1)^**

**I: Focus groups and interviews with autistic adults: headline topic guide**

| 1. **Welcome, general introductions, purpose and plan for the session, group rules** |
| --- |
| 1. **Round group introductions** |
| 1. **What information and support do people need after receiving their diagnosis?**   **‘Script’:** *We know that receiving any diagnosis is a major life event, even when people expect their diagnosis, and people can need information and support to understand their diagnosis, and come to terms with what it means for their everyday*  *We want to find out what information and support people need when they are given an autism diagnosis. To help us with us, we wondered if you could just take a moment to think back to when you were first given your diagnosis and tell us:*   - What did you want to know about your diagnosis at that stage? - Was there anything you did not want to know at this stage? - Did you want any other help at this stage? - Looking back, was there any other information or support you would have benefitted from in the first few months after your diagnosis? |
| **COMFORT BREAK** |
| 1. **The different ways in which information & support can be provided**   **‘Script’***: Provide brief overview what discussed before the comfort break before moving on to explore:*   - **Where did you go** to get your information and support? - Based on this, **what sources/people would you recommend**? - Do you think people should be offered **sessions with the team** who carried out the diagnosis to address these information and support needs? (*Ensure check for variation in views*) Why? If yes:   - - When should that be?     - Should these sessions be offered via a group or individually? Why? |
| 1. **Views on offering information and support via groups**   **‘Script’:** *Where services already run post-diagnostic groups. These are courses run for a group of people in which people are usually offered information about autism, as well as an opportunity to discuss their experiences with others. Some of you may have attended one of these courses. However, these courses are run in lots of different ways. We would like to know what you would prefer:*   - - **Staffing** - in some groups is run solely by staff who work in the diagnostic assessment service, others involve autistic adults. What would you recommend? Why?   - **Online** - due to the pandemic, some teams who already offer groups have had to move online. What do you think are the pros and cons of this?   - **Structure**–there is a service which offers those who receive an autism diagnosis with information about autism through a series of videos. Then, if people want to, they can come to a group to discuss the information provided in the video. What do you think about this? |
| 1. **Close**  - Any final comments/thoughts/reflections? - Thanks – appreciate that this may have brought back memories of difficult times which may not have thought about for a while. - Explain when findings will be available |

**II: Workshops with staff working in autism specialist services (professionals and experts by experience): workshop programme and headline topic guide for small group discussions**

| 1. Welcome. Purpose and structure of the workshop |
| --- |
| 1. Study participant introductions |
| 1. Researcher presentation: Initial findings from our research with autistic adults: experiences of the assessment process and feedback appointment (*including Q&A*) |
| 1. Small group discussion 1: Psychoeducation during the assessment process and feedback appointment  - Key discussion points:   - Information needs arising during the assessment process, and how to address.   - Adopting a positive / ‘strengths-focus’ approach to assessment and feedback.   - Information needs at the feedback appointment, and how to address. |
| *Break* |
| 1. Researcher presentation: Initial findings from our research with autistic adults: experiences of post-diagnostic support provided by the diagnostic service, including psychoeducation programmes |
| 1. Small group discussion 2: What makes a good group-delivered psychoeducation intervention?  - Key discussion points:   - Are groups positively good, or a pragmatic solution?   - How can groups address the need for ‘personalised psychoeducation’?   - The role of Experts by Experience in designing and facilitating psychoeducation interventions.   - Supporting engagement and take-up of group-delivered psychoeducation. |
| 1. Review of workshop discussions |
| 1. Next steps and close |

1. These headline topic guides offer an overview of the structure and content of the focus groups and interviews. More detailed versions were used by the research team [↑](#footnote-ref-1)
