# Supplementary material for: Incorporating Psychoeducational Care in the Autism Diagnosis Pathway: Experiences, Views, and Recommendations of UK Autistic Adults and Autism Professionals
Source: Autism Adulthood. 2025 Feb 5;7(1):13–24. doi: 10.1089/aut.2023.0060 (PMC11937777; doi:10.1089/aut.2023.0060)
Supplement: Supplementary Material S3 [file aut.2023.0060_suppl_materials3.docx]

**Supplementary Material 3:**

**Recommendations for psychoeducational care across the autism diagnosis pathway:**

**checklist for diagnostic services**

| **PAN-PATHWAY PYCHOEDUCATIONAL CARE AND PRACTICE** |
| --- |
| **‘CURATED’ INFORMATION RESOURCES** |
| - Diagnostic services should have a ‘curated’ collection of information on: living with autism, factual information about autism, and information about local, regional and national support organisations and services. The information resources recommended to individuals should be selected according to the stage on the diagnostic pathway and/or presenting information needs. |
| - Experts by experience should be closely involved in specifying and selecting content, and its ‘look and feel’. Different media or formats should be used, including text-based material, podcasts, videos, graphics/pictures. |
| **LANGUAGE/RHETORIC ABOUT AUTISM** |
| - Throughout, the language used should refer to difference rather than deficit. A person’s strengths and achievement should be noted, whilst acknowledging past or current difficulties. In one to one work, clinical judgement should be used to determine whether an overtly positive or balanced rhetoric should be used. In group work, a balanced rhetoric should be used. |
| **CLARITY ABOUT TIMING OF NEXT CONTACT** |
| - At all stages in the pathway where individuals are expecting to hear from the service (e.g. offer of an appointment, confirming dates for a psychoeducation programme), the likely timeline for next contact should be clearly explained. During waiting periods, autistic adults would value simple receiving ‘we’ve not forgotten you’ messages. |
| **PSYCHOEDUCATIONAL CARE AT DIFFERENT POINTS ON THE PATHWAY** |
| **THE ASSESSMENT SESSIONS** |
| - At an early stage in the diagnostic assessment process there should be a discussion with the individual about the different ways people respond to hearing the outcome of the assessment process. Clinicians should normalise these different emotional reactions. |
| - Where possible, and without compromising the assessment process, clinicians should explain the reason, or purpose, for each element of the assessment process. They should reassure individuals that fuller explanations will be provided at the feedback session. |
| - Information about the psychoeducational care offered by the service following an autism diagnosis should be provided early on in the assessment process, and reiterated towards the end of the assessment process. |
| - Clinicians should use assessment sessions to form an initial judgement on whether one-to-one delivery of psychoeducation programme is indicated, either due to the individual’s needs, or because of potential impacts on group dynamics or others attending the group. |
| - Details of selected ‘curated’ information sources should be shared. |
| **THE FEEDBACK SESSION** |
| - The feedback session should be a face-to-face |
| - It should include a clear explanation of how the team has come to the decision to diagnose autism, including the role played by the different components of the assessment process in coming to that decision. |
| - Clinicians should check for existing understandings of autism, exploring and correcting where required. |
| - Individuals should be asked about their feelings about the diagnosis and emotional reactions normalised. Clinicians should explain that feelings about the diagnosis may change over time. |
| - The session must include offering advice and discussing plans for disclosure. This should cover all relevant groups/settings (e.g work, family, social networks), including social media. |
| - The purpose of the assessment report(s) the individual will receive must be explained. |
| - Other elements of the post-diagnosis offer (e.g. de-brief appointment(s), psychoeducation programme) should be explained, including the ways these interventions will address the individual’s particular needs or situation. |
| - If a group-delivered programme is being offered, care should be taken to: i) recognise the challenges of attending a group; ii) explain why group-delivery is favoured); and iii) explain the autism-friendly/autism-specific accommodations in place to support attendance. Written and/or audio-visual (i.e. video) information should also be provided. This should cover programme content, photographs of facilitators and venue, and travel options. |
| - Details of selected ‘curated’ information sources should be shared. |
| **DEBRIEF APPOINTMENT(S)** |
| - Individuals should be offered at least one de-brief appointment with a member of diagnostic team in order to: to explore and discuss feelings about the diagnosis and emotional reactions to it, to support with making sense of the diagnosis, and to respond to questions about the diagnostic process or diagnosis itself. |
| - An appointment within 4 weeks of the feedback session should be offered, but with the option to request to delay. Where this occurs, clinics must undertake to pro-actively contact individuals to re-schedule the appointment. |
| - The appointment should check on experiences of disclosure and discussions and advice on managing any future disclosures. |
| - The individual should be reminded about the psychoeducation programme. Care should be taken to explain what the programme covers and the ways these interventions will address the individual’s particular needs or situation. |
| - If a group-delivered programme being offered, care should be taken to: i) recognise the challenges of attending a group; ii) explain why group-delivery is favoured); and iii) explain the autism-friendly/autism-specific accommodations in place to support attendance. Written and/or audio-visual (i.e. video) information should also be provided. This should cover programme content, photographs of facilitators and venue, and travel options. |
| - Details of selected ‘curated’ information sources should be shared. |
| **THE PSYCHOEDUCATION PROGRAMME** |
| - All adults (without significant learning disability) diagnosed with autism should be offered the opportunity to attend an autism psychoeducational programme. Those refusing the first offer should be offered the programme on at least one further occasion. |
| - Individuals will vary in terms of when they are ‘ready’ to attend a programme. Beforehand, there should be sufficient time for some degree of personal processing of the diagnosis to have occurred. However, long delays (i.e. several months) before attendance are likely to be unhelpful. |
| - Programmes should integrate didactic elements, personal reflection and learning from others (e.g. ‘personal account’ videos, others attending a group-delivered programme). Didactic elements should be kept to a minimum. |
| - An autism psychoeducation programme should cover the following topics:   - factual information about autism (e.g. diagnostic criteria; neurodiversity)   - understanding and identifying how autism impacts me as an individual, and what it means for my life   - sharing and exploring emotional responses to the diagnosis   - learning self-management skills, particularly managing anxiety, disclosure, self-care, self-advocacy, and sourcing trustworthy information   - information and advice on rights and entitlements   - supporting connections with other autistic people, including other group members |
| - Workbooks/handouts directly related to session content is valued. Details of selected ‘curated’ information sources should also be shared. |
| - Group-delivery should be the routine mode of delivery, but with the option of one to one sessions available. |
| - Groups should be co-delivered by clinicians and experts by experience. |
| - Where possible, limit the size of the group to 8 or less, and offer groups in the evenings as well as during the day. |
| - Venues used for group programmes should be easily accessible. Consider using community spaces rather than where the service is based. Provide clear information (including photographs) of the venue and travel directions. |
| - A number of autism specialist services have developed psychoeducational programmes. However, none have been published or externally evaluated. Services considering introducing a psychoeducation programme should consider using or adapting an existing programme. Autistic adults should be closely involved in selecting and adapting existing programmes. |
